# The “Special” *crystal-Stellate* System in *Drosophila melanogaster* Reveals Mechanisms Underlying piRNA Pathway-Mediated Canalization

**DOI:** 10.1155/2012/324293

**Published:** 2011-12-15

**Authors:** Maria Pia Bozzetti, Laura Fanti, Silvia Di Tommaso, Lucia Piacentini, Maria Berloco, Patrizia Tritto, Valeria Specchia

**Affiliations:** ^1^Dipartimento di Scienze e Tecnologie Biologiche ed Ambientali, Università del Salento, 73100 Lecce, Italy; ^2^Sezione di Genetica, Dipartimento di Biologia e Biotecnologie “Charles Darwin”, Sapienza Università di Roma, 00185 Roma, Italy; ^3^Dipartimento di Biologia, Università degli Studi di Bari Aldo Moro, 70121 Bari, Italy

## Abstract

The Stellate-made crystals formation in spermatocytes is the phenotypic manifestation of a disrupted *crystal-Stellate* interaction in testes of *Drosophila melanogaster. Stellate* silencing is achieved by the piRNA pathway, but many features still remain unknown. Here we outline the important role of the *crystal-Stellate* modifiers. These have shed light on the piRNA pathways that defend genome integrity against transposons and other repetitive elements in the gonads. In particular, we illustrate the finding that HSP90 participates in the molecular pathways of piRNA production. This observation has relevance for the mechanisms underlying the evolutionary canalization process.

## 1. The Stellate-Made Crystals in Spermatocytes Are the Phenotypic Manifestation of a Disrupted *crystal-Stellate* Interaction in Testes of *Drosophila melanogaster*

The history of the *crystal-Stellate* system started in 1961 when Meyer and collaborators discovered the presence of crystalline aggregates in primary spermatocytes of *D. melanogaster X/O* male testes. They also described the morphological differences between needle-shaped and star-shaped crystals [1].

In 1983, Gatti and Pimpinelli provided a detailed cytological description of the *Y* chromosome. They showed that the *hll* region contains the genetic determinants for normal chromosome behavior during male meiosis and for the suppression of Stellate-made crystals formation in spermatocytes [2]. This region was called the *Suppressor of Stellate* [*Su*(*Ste*)] locus, also referred to as *crystal* (*cry*) [3]; in this paper we use “*crystal*.”

Afterwards, different groups established that both the morphology of the crystalline aggregates and the severity of the meiotic defects in *X/O* and *X*/*Y* ^cry-^ males depend on the *Stellate *(*Ste*) locus on the *X* chromosome [4–6]. Two regions containing clustered *Stellate* elements have been identified on the *X* chromosome: *12E1* in euchromatin and *h27 *in heterochromatin. *Stellate* and *crystal* are both repetitive sequences and they share sequence homology [6–8].

**Figure 1:**
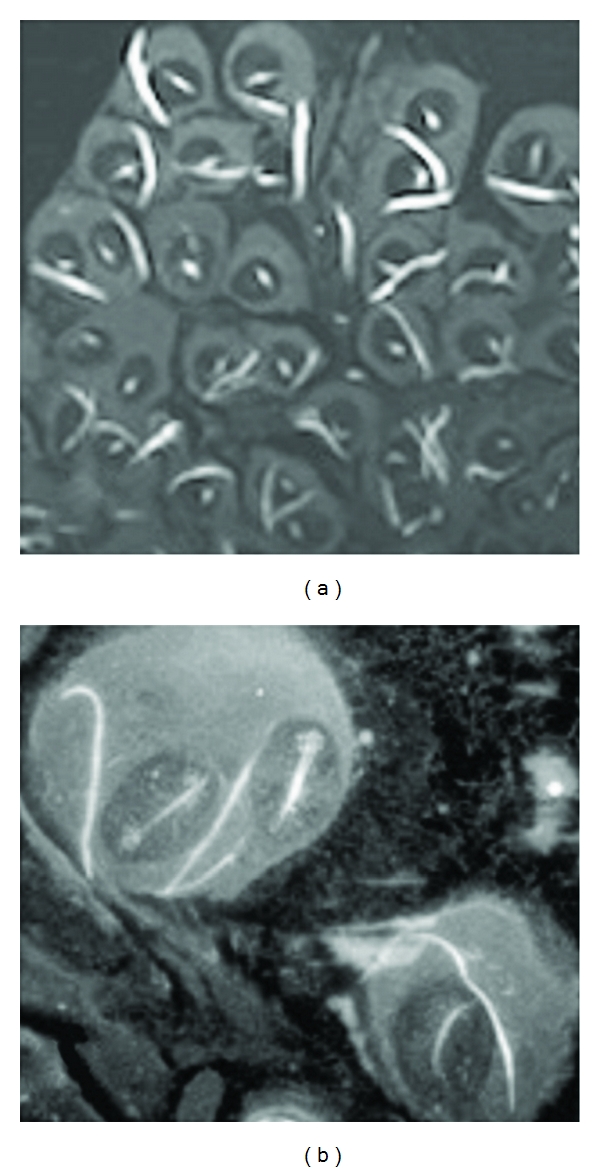
Testes of *X*/*Y* ^cry-^ males immunostained with anti-Stellate antibody, (a) magnification 20x; (b) magnification 40x.

At the molecular level, the loss of the *crystal* region results in the production of a testes-specific *Stellate* mRNA of 750 nucleotides in length. The product of this mRNA is the Stellate protein [8, 9]. In 1995 there was a fundamental discovery: the Stellate protein is the main component of the crystals in the primary spermatocytes [10] and Figure 1.

## 2. The Regulation of the *crystal-Stellate* Interaction

The first indication about the mechanism that regulates the interaction between *crystal *and *Stellate *sequences was obtained in 2001; the *Stellate *silencing was associated with the presence of small RNAs, 24–29 nt long, homologous to *crystal* and *Stellate* sequences [11]. These were named rasiRNAs (repeat-associated small interfering RNAs) [12].

The detailed analysis of the *crystal*-rasiRNAs in fly testes demonstrated the existence of a specific RNAi pathway in the germline that silences repetitive sequences such as Stellate and transposable elements [13]. It was also demonstrated that rasiRNAs show differences in structure compared to other classes of small noncoding RNAs, such as siRNAs and miRNAs and their biogenesis is Dicer-independent [13]. The rasiRNAs work associated with the Piwi subfamily of the Argonaute proteins, Aubergine, Ago3, and Piwi. rasiRNAs were subsequently designated as Piwi-interacting RNAs or piRNAs [13]. The studies on the *crystal-Stellate* system have been therefore crucial for the discovery of the piRNA pathway.

In 2007, two independent groups used a deep sequencing strategy to identify small RNAs bound to each of the three Piwi proteins in fly ovaries. Their expectation was that this approach would reveal how piRNAs were made and how they function. They demonstrated that piRNAs arise from a few genomic sites, grouped in clusters that produce small RNAs that silence many transposons [14, 15]. In fly testes, the most abundant Aubergine-associated piRNAs (~70%) correspond to *crystal *antisense transcripts [16].

## 3. The piRNA Pathways in the Fly Ovaries

Studies on the sequences of the small RNAs associated to Piwi subclade proteins carried out in 2006 and 2007 by the Hannon, Zamore, and Siomi groups have been crucial to formulation of a model for the biogenesis and the function of the piRNAs in the germline [13–16]. The proposed model, called the “ping-pong” model, requires a primary piRNA, whose biogenesis has not yet been elucidated, bound by Aubergine or Ago3. In particular, Aub binds an antisense piRNA and cleaves the sense transcript from an active transposon; transcript cleavage produces a sense piRNA that is loaded onto Ago3. This Ago3-piRNA complex binds complementary transcripts and initiates the production of piRNAs by an amplification loop [14]. The piRNAs originated by this mechanism are now called “secondary” piRNAs and they exhibit specific signatures consisting of the adenine at the 10th position of the sense piRNAs, which is able to base pair with the initial uracil of the antisense piRNAs [14, 15].

Identification of *ago3* mutants led to the discovery of two different piRNA pathways in the fly ovary: one in the somatic cells of the ovary and the other in the germline cells. The somatic pathway, called “primary piRNA pathway,” involves Piwi, and it does not require an amplification loop. This pathway regulates the transposons belonging to the so-called “somatic” group [17, 18].

## 4. The piRNA Pathways in Fly Testes and Open Questions

Deep sequencing of piRNAs bound to Piwi-subfamily proteins associated to genetic studies, supplied thousands of data about almost all the piRNAs sequence biogenesis and orientation produced in testes [16, 19].

**Figure 2:**
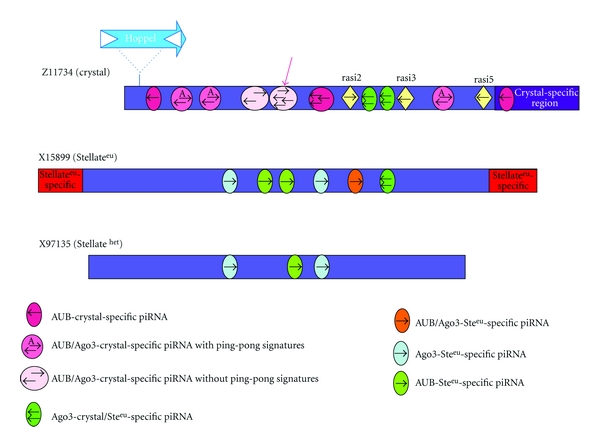
Schematic of the elements of the *crystal-Stellate* system. *crystal* (corresponding to sequence Z11734); euchromatic *Stellate* (corresponding to sequence X15899); heterochromatic *Stellate* (corresponding to sequence X97135). The position and the orientation of the most prominent piRNAs is indicated, on each element, by the colored little circles and rumbles. The sequence and the length of indicated piRNAs can be deduced from the Supplemental Figure 1. The Piwi protein to which each is bound is also indicated. The drawing is schematic and not to scale.

Although the overall structure of the *crystal *and *Stellate* loci remains unclear, regions of homology between *crystal* and *Stellate* piRNAs, and repeat monomers from each of these loci has been summarized in the scheme depicted in Figure 2. The position of several piRNAs on the *crystal* and *Stellate *sequences, their orientation and the Piwi protein(s) to which they are bound are indicated. Detailed information on the sequences of *crystal* (Z11734) and *Stellate *euchromatic sequences (X15799), depicting the location of piRNAs, are shown in Figure 1S (see Figure 1S in supplementary material available online at doi:10.1155/2012/324293). In light of this map we note that almost all the *crystal*-specific piRNAs come from the region, depicted in purple, of homology with *Stellate *sequences. These are predominantly “antisense” as already reported [11, 12, 14, 16, 19]. However, *Stellate*-specific piRNAs, whether euchromatic or heterochromatic, are predominantly in the “sense” orientation (Figure 2).

The majority of these piRNAs do not show the ping-pong signature. There are only 3 pairs exhibiting the A at the 10th position of the “sense” piRNA, and these “sense” piRNAs show 2 or 3 mismatches with *Stellate* euchromatic and heterochromatic sequences. Therefore, they cannot be considered canonical ping-pong pairs [Figure 1S(a)]. The *crystal*-specific piRNA, reported to be the most abundant one in testes, is only antisense [19], Figure 2.

For all the reasons reported above, we hypothesize that different though interconnected pathways exist to silence *crystal *and *Stellate* sequences. *crystal- *and* Stellate-*specific piRNAs cooperate in some way to silence the *Stellate* euchromatic and heterochromatic sequences that produce the Stellate protein (“active elements”) [10, 20]. These different pathways could be present in both the somatic and germline tissues of testes.

In support of these considerations, we refer to the previous data on the silencing of another class of repetitive sequences in testes. In fact, a second large class of piRNAs associated with Aubergine in the testes is derived from a short repeated region, termed *AT-chX*, on the X chromosome [16]. These piRNAs are predominantly antisense. Only one pair with ping-pong signatures was found among all sequenced *AT-chX* piRNAs. These remarks confirm that the ping pong is not the only piRNA pathways operating in the silencing of these repetitive sequences in testes [19].

## 5. Mutants Affecting the *crystal-Stellate* Interaction Clarify Unknown Aspects of the piRNA Pathways in Testes

Mutations in piRNA-pathway genes, such as *aubergine*, *ago3*, *spindle E*, *armitage*, *zucchini*, and *squash*, lead to the formation of the Stellate-made crystals in spermatocytes [17, 21–24].

*spindle-E *encodes a member of the DExH family of ATPases with a Tudor domain. Mutations in this gene are known to impair *Stellate* and transposon silencing in the *Drosophila* germline. In ovaries *spindle-E* acts specifically in germ cells and in the ping-pong cycle [18, 22, 25].

*Armitage *encodes a homolog of the *Arabidopsis* SDE3, an RNA helicase that is involved in RNAi. Mutations in *armitage *affect translational repression and localization of *oskar *mRNA, block RNAi in *Drosophila* oocytes, and impair *Stellate* silencing in testes [23, 26]. In ovaries, *armitage* acts in the primary piRNA pathway [18, 27, 28].* zucchini *was identified in a screen for female sterile mutations, and causes dorsoventral patterning defects. This gene encodes a nuclease. Mutations in *zucchini *lead to formation of Stellate crystals [24]. In ovaries* zucchini *mutations specifically decrease the piRNA levels in somatic ovarian cells [18].

In Table 1, we listed some of the modifiers of the *crystal-Stellate* interaction that have been related to the piRNA pathways in gonads. Mutants of genes implicated either in the primary piRNA pathway, excepting *piwi*, or in the secondary ping-pong amplification pathway show crystals in their spermatocytes.

After all, we are convinced that the molecular mechanisms, underlying the piRNA pathways, are not completely understood and that there are more players to be discovered in both the somatic and germline-specific piRNA pathways. The genetic characterization of known and still unknown components, combined with the deep sequencing strategy of the piRNAs bound to specific Piwi proteins, will help us in understanding the piRNAs production and function in the *Drosophila* testes. Because Stellate-made crystals are symptomatic of a disrupted *crystal-Stellate* interaction, they allow the identification of new genetic components of the piRNAs pathway. An emblematic example is the discovery that the *hsp83 *gene participates in piRNA.

## 6. *hsp83* ^**scratch**^, an Unexpected *crystal-Stellate* Modifier

The* hsp83* gene encodes HSP90 protein, a molecular chaperone involved in several cellular processes and developmental pathways [30–33]. We have recently demonstrated that primary spermatocytes of *hsp83^scratch^*  homozygous mutant males exhibit Stellate-made crystalline aggregates, suggesting a role for this protein in the piRNA-mediated mechanisms. We also demonstrated that *hsp83^scratch^*  affects the biogenesis of the *crystal/Stellate*-specific piRNAs and transposon piRNAs in testes. We went on to demonstrate that the effect of HSP90 in morphological variations is due, at least in part, to activation of transposons causing *de novo* mutations [29]. Among the *hsp83* mutant flies showing morphological abnormalities, we selected one exhibiting a *Scutoid*-like phenotype and demonstrated that this phenotype is caused by the insertion of an *I* element*-*like transposon in the *noc *gene of this fly.

The role of HSP90 in piRNAs-mediated silencing is in addition to the “buffering” role on the genetic cryptic variation initially put forth by Rutherford and Lindquist [34] as the molecular explanation for the Waddington's “canalization” process.

Canalization is the resistance of an organism to phenotypic variation during development, in the presence of genetic and environmental changes. This “phenotype robustness” is due to buffering mechanisms. Severe perturbations, which reduce buffering, produce heritable phenotypic variants that can be canalized by a genetic assimilation process [35]. An interesting aspect to investigate is if, and how, the reduction of HSP90 causes a stress response-like activation of mobile elements, creating a link between environmental changes and genomic variation.

Further mechanisms could be involved in increasing the phenotypic variations underlying evolution. One of these could be related to HSP90-mediated epigenetic chromatin modifications [36, 37].

## Supplementary Material

Precise localization of specific piRNAs on the Stellate (X15899) and crystal (Z11734) sequences. In order to visualize the region of the Stellate and crystal sequences that share homology with the most abundant piRNAs related to them (Nagao et al. 2010), we precisely localized some of these piRNAs on the sequences.
We selected two emblematic sequences for Stellate (X15899) and crystal (Z11734). We also reported their orientation and the Argonaute proteins which the reported piRNAs are bound to.

## Figures and Tables

**Table 1:** List of some genes involved in the piRNA pathways.

Genes	Crystals	Function	Ping pong*	References
*Aubergine*	+	Piwi protein	− −/+	[14–19, 21]
*Ago3*	+	Piwi protein	− −/+	[17–19]
*Piwi*	−	Piwi protein	+	[13–18]

*Spindle-E*	+	RNA helicase	− −/+	[18, 22, 25]
*Squash*	+	Tudor-domain nuclease	+	[24]

*Zucchini*	+	Nuclease	+	[24]
*Armitage*	+	RNA helicase	+	[18, 23, 26–28]

*hsp83*	+	Heat-shock protein	nd	[29]

*“+” indicates that the ping pong is functional in the mutant.

## References

[1] Meyer G, Hess O, Beermann W (1961). Phasenspezifische funktionsstrukturen in spermatocytenkernen von *Drosophila melanogaster* und ihre abhangigkeit vom Y chromosom. *Chromosoma*.

[2] Gatti M, Pimpinelli S (1983). Cytological and genetic analysis of the *Y* chromosome of *Drosophila melanogaster*. I. Organization of the fertility factors. *Chromosoma*.

[3] Pimpinelli S, Bonaccorsi S, Gatti M, Sandler L (1986). The peculiar genetic organization of Drosophila heterochromatin. *Trends in Genetics*.

[4] Hardy RW, Lindsley DL, Livak KJ (1984). Cytogenetic analysis of a segment of the Y chromosome of *Drosophila melanogaster*. *Genetics*.

[5] Livak KJ (1984). Organization and mapping of a sequence on the *Drosophila melanogaster* X and Y chromosomes that is transcribed during spermatogenesis. *Genetics*.

[6] Palumbo G, Bonaccorsi S, Robbins LG, Pimpinelli S (1994). Genetic analysis of *Stellate* elements of *Drosophila melanogaster*. *Genetics*.

[7] Tulin AV, Kogan G, Filipp D, Balakireva MD, Gvozdev VA (1997). Heterochromatic Stellate gene cluster in *Drosophila melanogaster*: structure and molecular evolution. *Genetics*.

[8] Tritto P, Specchia V, Fanti L (2003). Structure, regulation and evolution of the *crystal-Stellate* system of Drosophila. *Genetica*.

[9] Livak KJ (1990). Detailed structure of the *Drosophila melanogaster* Stellate genes and their transcripts. *Genetics*.

[10] Bozzetti MP, Massari S, Finelli P (1995). The *Ste* locus, a component of the parasitic *cry-Ste* system of *Drosophila melanogaster*, encodes a protein that forms crystals in primary spermatocytes and mimics properties of the *β* subunit of casein kinase 2. *Proceedings of the National Academy of Sciences of the United States of America*.

[11] Aravin AA, Naumova NM, Tulin AV, Vagin VV, Rozovsky YM, Gvozdev VA (2001). Double-stranded RNA-mediated silencing of genomic tandem repeats and transposable elements in the *D. melanogaster* germline. *Current Biology*.

[12] Aravin AA, Lagos-Quintana M, Yalcin A (2003). The small RNA profile during *Drosophila melanogaster* development. *Developmental Cell*.

[13] Vagin VV, Sigova A, Li C, Seitz H, Gvozdev V, Zamore PD (2006). A distinct small RNA pathway silences selfish genetic elements in the germline. *Science*.

[14] Brennecke J, Aravin AA, Stark A (2007). Discrete small RNA-generating loci as master regulators of transposon activity in drosophila. *Cell*.

[15] Gunawardane LS, Saito K, Nishida KM (2007). A slicer-mediated mechanism for repeat-associated siRNA 5' end formation in Drosophila. *Science*.

[16] Nishida KM, Saito K, Mori T (2007). Gene silencing mechanisms mediated by Aubergine-piRNA complexes in Drosophila male gonad. *RNA*.

[17] Li C, Vagin V, Lee S (2009). Collapse of germline piRNAs in the absence of argonaute 3 reveals somatic piRNAs in flies. *Cell*.

[18] Malone CD, Brennecke J, Dus M (2009). Specialized piRNA pathways act in germline and somatic tissues of the Drosophila ovary. *Cell*.

[19] Nagao A, Mituyama T, Huang H, Chen D, Siomi MC, Siomi H (2010). Biogenesis pathways of piRNAs loaded onto AGO3 in the Drosophila testis. *RNA*.

[20] Kotelnikov RN, Klenov MS, Rozovsky YM, Olenina LV, Kibanov MV, Gvozdev VA (2009). Peculiarities of piRNA-mediated post-transcriptional silencing of Stellate repeats in testes of *Drosophila melanogaster*. *Nucleic Acids Research*.

[21] Schmidt A, Palumbo G, Bozzetti MP (1999). Genetic and molecular characterization of sting, a gene involved in crystal formation and meiotic drive in the male germ line of *Drosophila melanogaster*. *Genetics*.

[22] Stapleton W, Das S, McKee B (2001). A role of the Drosophila *homeless* gene in repression of *Stellate* in male meiosis. *Chromosoma*.

[23] Tomari Y, Du T, Haley B (2004). RISC assembly defects in the Drosophila RNAi mutant armitage. *Cell*.

[24] Pane A, Wehr K, Schüpbach T (2007). Zucchini and squash encode two putative nucleases required for rasiRNA production in the Drosophila germline. *Developmental Cell*.

[25] Lim AK, Kai T (2007). Unique germ-line organelle, nuage, functions to repress selfish genetic elements in *Drosophila melanogaster*. *Proceedings of the National Academy of Sciences of the United States of America*.

[26] Cook HA, Koppetsch BS, Wu J, Theurkauf WE (2004). The Drosophila SDE3 homolog armitage is required for oskar mRNA silencing and embryonic axis specification. *Cell*.

[27] Olivieri D, Sykora MM, Sachidanandam R, Mechtler K, Brennecke J (2010). An in vivo RNAi assay identifies major genetic and cellular requirements for primary piRNA biogenesis in Drosophila. *The EMBO Journal*.

[28] Saito K, Ishizu H, Komai M (2010). Roles for the Yb body components Armitage and Yb in primary piRNA biogenesis in Drosophila. *Genes and Development*.

[29] Specchia V, Piacentini L, Tritto P (2010). Hsp90 prevents phenotypic variation by suppressing the mutagenic activity of transposons. *Nature*.

[30] Ding D, Parkhurst SM, Halsell SR, Lipshitz HD (1993). Dynamic Hsp83 RNA localization during Drosophila oogenesis and embryogenesis. *Molecular and Cellular Biology*.

[31] Cutforth T, Rubin GM (1994). Mutations in Hsp83 and cdc37 impair signaling by the sevenless receptor tyrosine kinase in Drosophila. *Cell*.

[32] Hartl FU (1996). Molecular chaperones in cellular protein folding. *Nature*.

[33] van der Straten A, Rommel C, Dickson B, Hafen E (1997). The heat shock protein 83 (Hsp83) is required for Raf-mediated signalling in Drosophila. *EMBO Journal*.

[34] Rutherford SL, Lindquist S (1998). Hsp90 as a capacitor for morphological evolution. *Nature*.

[35] Waddington CH (1942). Canalization of development and the inheritance of acquired characters. *Nature*.

[36] Sollars V, Lu X, Xiao L, Wang X, Garfinkel MD, Ruden DM (2003). Evidence for an epigenetic mechanism by which Hsp90 acts as a capacitor for morphological evolution. *Nature Genetics*.

[37] Gangaraju VK, Yin H, Weiner MM, Wang J, Huang XA, Lin H (2011). *Drosophila* Piwi functions in Hsp90-mediated suppression of phenotypic variation. *Nature Genetics*.

