# Supplementary material for: The “Special” crystal-Stellate System in Drosophila melanogaster Reveals Mechanisms Underlying piRNA Pathway-Mediated Canalization
Source: Genet Res Int. 2011 Dec 15;2012:324293. doi: 10.1155/2012/324293 (PMC3335654; doi:10.1155/2012/324293)
Supplement: Supplementary file 1 — Precise localization of specific piRNAs on the Stellate (X15899) and crystal (Z11734) sequences. In order to visualize the region of the Stellate and crystal sequences that share homology with the most abundant piRNAs related to them (Nagao et al. 2010), we precisely localized some of these piRNAs on the sequences. We selected two emblematic sequences for Stellate (X15899) and crystal (Z11734). We also reported their orientation and the Argonaute proteins which the reported piRNAs are bound to. [file 324293.f1.pdf]

1 aacttattat gaaataaaag aactaatact tattatgcca gccgaacata aaacgaattt tcgagtctag agttcccatc tgcaagggca tgacagagtc
101 ctggcaaaga cactagaata acaagatgca taacgaccat acattggttt ggcactatgc agccactttt ttagtgacgg ccaaaattgc tctctttcca
201 ctcgctcacg ctgagagcgt aagaaatcta aaaatagaat ttgcttgctt gtgtgagtaa aaacaagaga cgagaacgcg tacgcgtata tgtgtgcgtg
301 ttgtggtaga cgattttcgg gccgaaataa attctgatcg aagaaacgaa tttatactgt acatattagg gtagtttttt ccaatttcgt agcaatatga
401 taaaataaaa taatttttaa aaaaattaaa gctttttaaa ttcgtttctt aaaatcgccg ctcgaattag ctaccgttta cacatttata tttatgttta
501 attctaattt gtctctcatc tgacaatctt ttaaaagcga aatatttttt tgaaacactt ttaatcttaa tgttacatga tattaagtca aatgacttaa
601 taaatgtact aaataattta aggaagtaca atagaaatta ttatagctac tgtaatttca taaattttct aaaaagaaga cattacgttg agaaataaat
701 atttcataaa aataaattta taaaataaat aaacatataa cggtttattg caaaagtcat atcaaaggca ctagaattat tctagtgtct ttgctttggt
801 catatcttga ggcacgaagt gcggacacaa gcactcaaca attattgcct aattattttt tcacacgacg caagatcaat actctaatga caaatattct
901 tatatagtca ttttttaaat ttattttgtg ataatatgta catagatttg gctatttcta atctattttc aaataataat aacgttaagg caatgcaaaa
1001 caagaatttt tcgcatggtg ccaattgatc aaaaataata tagatttaaa gtctaagaac ttctgaggtg aagggcatat tttgtcaaat ttatatacat
1101 acagctgtct gctatcactg tatgcgcaga agtgctgttc gctgtagcgc tctccgctct ctcgctctct aacaaaaatt cgagagagcc tggagccacc
1201 tctagagcca cggcgaaaaa atcgtatggc gttatgcatc ttattatttt agtgtctttg gtcctgtcaa cccttttagc aagtgtcaaa aactcaaaga

AUB-crystal-specific piRNA(-)

1301 aaaagacgat gactttgaag tctacaagtc atatttctgt gaacgagtga actggcaac**A TG**tcga***gcct gtaagtaact aggtttttcc*** tatagaaatt

AUB/AGO3**-crystal-specific piRNA(-)22x** AUB/AGO3-crystal-specific piRNA(+)4x

1401 atagcaagtc acagtaaaat cttgaacaca gaacaacaac aacaaca***gca gctggatcaa ttggttcctc gga***Atcaagg gcaacgagtt cctctgccac

AUB/AGO3**-crystal-specific piRNA(-)7x** AGO3 crystal-specific piRNA(+)3x **rasi1 (-)** **rasi4 (-)**

1501 gtgccca***cca actactttca ggacacgttc a***accagAtgg gcctggagta cttcagccag cactggacgt gatc***ctgaag ccggcgttcg acagttcctc***

very abundant crystal-specific
AUB/AGO3 piRNAs (-) thousands x

1601 ***gg****gcttgttc tacgacgatg aga*aaaagtg gtacggcatg attcacgccc gatacatcag gtcggagcgt ggcgtgaatg atatgcaccg aaactatatg

AGO3- piRNA(+) Region producing many different crystal-specific AUB-piRNAs

1701 agaggagact ttgaatcgtg tccgaatatc tcctgtaata ggaagaaca***c cctgccagtg ggcctcagcg atgtgtgggg caagtcaacg gtgaagatct***

rasi2 (+)

1801 ***actgccca***cg ctgtaaaaac tacttccatc ctaaaactga tacacagcc***a tgttcgagcc cagcttcccg gaca***tcttct taacgcagct g***ccgaacttg***

AGO3-crystal-specific piRNAs(-)(hundreds) AGO3-crystal/Stellate-specific piRNAs (-)11x

1901 ***agaccgcccc tggacgacc***c acggtaagta attctc***cgaa tatagtcctg gttgttttct a***aacaaagcg cttgaacttg cagtccctat gctttctgtt

rasi3 (-)

2001 ***gcaccagaag gccttgatgc cact***taaatc gcccaagtcc tcaccaaaga aaatcggatc ctcggccagc tagtcctcct cctcgccccg gcattccgaa

AUB-Su (Ste)4 (-)15 x AGO3-piRNA(+)

2101 ***gtcaagcgct tcaatgttct tctA***gggatg acgtgatttt ttaactccac acaaaagaaa gcagaaatgt ttgtccgata tattgttgtt gcaattaaac

rasi5 (-)

2201 agattccgca gtgcgttgat atggccagct ***accacctact aatatacctc ata***cacctct cctctaaata cttggttcaa agtgttcggt ccaaactggt

AUB-crystal-specific piRNA(-)102 x

2301 catatcaagc actcattcga gtagcaaagc atacttttcg ttaatagtgg taatcacctg ggtataataa taatattaat caataaaaca ttcaattcag
2401 ttttttattt aattttatgt taaaagagtt tccaaaagtg ttttcctgac tacatcaaat atttttttgat aatttaattt ctttaaatat acaaattctg

2501 ttttaatgta ttataggagt gagtagaagg ttaaaacatt attttcaatt tcaaataaaa ttaaaatttt gattccatca tttttatgta attctttttg
2601 tagagtattc aatactggca acttcgtatt ttagtttgat ttcccatgct gtttttggtt aatttccaac ataccagatt gatttcgcgc gtaaatttaa
2701 acgcacttta ttgccttttt ggaagaacgt tgcttcaaaa cggaaaatcg aaggaaaccc tgaccggacc taagggacag ccttgggggg aaaactggca
2801 gtgcattttc ccagccccaa agattttccc cggcaagtcg ggg
