# Supplementary material for: The “Special” crystal-Stellate System in Drosophila melanogaster Reveals Mechanisms Underlying piRNA Pathway-Mediated Canalization
Source: Genet Res Int. 2011 Dec 15;2012:324293. doi: 10.1155/2012/324293 (PMC3335654; doi:10.1155/2012/324293)
Supplement: Supplementary file 2 [file 324293.f2.pdf]

Ste^{het} start

1 aattcagttt tctttaattt taattaattt ttttctaaga aaaataaata aaaacgaaaa attcaaataa aaattataaa aaataaaccg agttgtgttt

101 tatttcacgt taaatagtta agtggtaata taactgaatg gtaagtttat taattaattt aagtgttaaa aaattactga atttatagga ttttgatttg

201 gatactattt ataaaatcat ataaaaggcc atcgagtcct cagccgatgc tgccttgtcg aaacaatgac atttgatttg tttttggccc aactgacaca

301 taaaatatcg tatgcataac atattatgaa ataaaagaac taatacttat tatgccagcc gaacataaaa cgaattttcg agtctagagt tcccatctgg

hoppel insertion in *crystal*

401 aagggcaggc cttttagcac gtgtcaaaaa ctcaaagaag aagacgatga ctttgaagtc tacaagtcat atttctgtga tcaagtgaac tggcaac**ATG**

501 tcgagctcGT AAGTAACTAG GTTTTTCCTA TAGAAATTAT AGCAAGTCAC AGTAAAATCT TGTAGccaga acaacaacag cagctggatc gattggttcc

AGO3-.Ste^{eu}/Ste^{het} specific piRNA(+)

601 tcgggatcaa gggcaaccag ttcctctgcc gcgtgcccac cgactacgtg caggatacgt tcaaccagat gggcttggag tatttcagcg agatactgga

AUB- Ste^{eu} specific piRNA (+)

AUB- Ste^{eu} specific piRNA (+)

701 cgtgatcctg aagccggtga tcgatagttc ctctggcttg ttgtacggcg atgaaaagaa gtggtacggc atgattcacg cccgatacat caggtcagag

AGO3-Ste^{het} specific piRNA (+)

801 cgtggcctga ttgctatgca ccgaaaatat ttgcgaggag attttggatc gtgtcccaat atctcctgtg ataggcagaa caccctgcca gtgggcctca

AGO3-Ste^{eu}/Ste^{het} specific piRNA (+)

901 gcgctgtatg gggcaagtca accgtcaaga tccactgccc acggtgtaaa agcaactttc atccgaagtc tgatacacag ctggacggag cgatgttcgg

AUB/AGO3-Ste^{eu} specific piRNA(+)

AGO3-crystal-Ste^{eu} piRNA(-)11X

1001 gcccagcttc ccggacatct tcttctcgct gctgccgaac ttgacatcgc ccctggacga cccacggtaa GTAATTCTCC GAATATAGTC CTGGTTGTTT

STOP

Ste^{het} end

1101 TCTAAACAAA GCGCTTGCAC TTGCAGtacc taggctttcg gttgcaccaa agagctttga tgcaactcaa atcgccccaa gccaaagaata tcggaatatt

1201 ggtcaggcgc atcaatgtat ttttgtatgt atattgttgt tgttgtaatc aatacgaaaa atcaagaaca tattc
